# IGF2BP2 Alternative Variants Associated with Glutamic Acid Decarboxylase Antibodies Negative Diabetes in Malaysian Subjects

**DOI:** 10.1371/journal.pone.0045573

**Published:** 2012-09-19

**Authors:** Sameer D. Salem, Riyadh Saif-Ali, Ikram S. Ismail, Zaid Al-Hamodi, Rozaida Poh, Sekaran Muniandy

**Affiliations:** 1 Department of Molecular Medicine, Faculty of Medicine, University of Malaya, Kuala Lumpur, Malaysia; 2 Department of Biochemistry, Faculty of Medicine, Sana’a University, Sana’a, Yemen; 3 Department of Medicine, Faculty of Medicine, University of Malaya Medical Centre, University of Malaya, Kuala Lumpur, Malaysia; Tehran University of Medical Sciences, Islamic Republic of Iran

## Abstract

**Background:**

The association of Insulin-like growth factor 2 mRNA-binding protein 2 (IGF2BP2) common variants (rs4402960 and rs1470579) with type 2 diabetes (T2D) has been performed in different populations. The aim of this study was to evaluate the association of alternative variants of IGF2BP2; rs6777038, rs16860234 and rs7651090 with glutamic acid decarboxylase antibodies (GADA) negative diabetes in Malaysian Subjects.

**Methods/Principal Findings:**

IGF2BP2; rs6777038, rs16860234 and rs7651090 single nucleotide polymorphisms (SNPs) were genotyped in 1107 GADA negative diabetic patients and 620 control subjects of Asian from Malaysia. The additive genetic model adjusted for age, race, gender and BMI showed that alternative variants; rs6777038, rs16860234 and rs7651090 of IGF2BP2 associated with GADA negative diabetes (OR = 1.21; 1.36; 1.35, P = 0.03; 0.0004; 0.0002, respectively). In addition, the CCG haplotype and diplotype CCG-TCG increased the risk of diabetes (OR = 1.51, P = 0.01; OR = 2.36, P = 0.009, respectively).

**Conclusions/Significance:**

IGF2BP2 alternative variants were associated with GADA negative diabetes. The IGF2BP2 haplotypes and diplotypes increased the risk of diabetes in Malaysian subject.

## Introduction

Diabetes mellitus is a quickly emergent public health problem with a tremendous impact on morbidity and premature mortality worldwide. It is a complicated heterogeneous metabolic disorder that affects 366 million people worldwide (6.4% of the world’s adult population aged between 20–79 years). This number will be increased to 552 million by 2030 [1]. More than 2.03 million of Malaysians have diabetes mellitus (11.66% of the Malaysian’s adult population) [1]. Among T2D patients, Latent Autoimmune Diabetes of Adults (LADA) occurs in 2.8–22.3% of individuals [2], [3], [4]. LADA can be distinguished with evidence of GADA positivity in adult diabetic patients who clinically resemble T2D at diagnosis [2], [5], [6] and do not initially require insulin for at least 6 months [7].

It is widely accepted that interactions between multiple environmental and genetic factors contribute to the initiation and progression of T2D [8], [9], [10]. Several genes those are associated with T2D in more than one population. However, subsequent association studies showed some variants that were highly associated with T2D in one group of subjects were not associated in other. GWA studies on Caucasians revealed that IGF2BP2 is a candidate risk gene for T2D [11], [12], [13], [14], [15], [16], [17], [18].

The majority of subsequent association studies of IGF2BP2 with T2D were focused on intron 2 SNPs; rs4402960 and rs1470579 [10], [13], [19], [20], [21], [22], [23], [24], [25], [26], [27], [28], [29]. It is becoming clear that single susceptibility locus is not shared among all ethnic groups. In addition, haplotype variation in populations may indicate different SNPs within IGF22BP2 gene that may be associated with T2D, which might be located at a distance downstream or upstream of the most common variants. However, there are limited studies on the association of IGF2BP2 alternative variant; rs6777038, rs16860234 and rs7651090. The aim of this study was to evaluate the association of alternative variations within IGF2BP2 gene with GADA negative diabetes in Malaysian subjects.

## Materials and Methods

### Ethnic Statement

This study was approved by the local Medical Ethics Committee of University Malaya Medical Centre. Written informed consent was obtained from each participant.

### Subjects and Data Collection

Case-control study was conducted at the University Malaya Medical Centre (UMMC) Kuala Lumpur. The patients previously diagnosed with T2D who attended the UMMC for treatment were invited to participate in this study (case group). For the control group, subjects who were enrolled for health check-up at UMMC were approached to participate in this study. All participants enrolled in this study were Malaysian aged 30–70 years in which non-probability sampling was used. Venus blood (10ml) was collected from each participant after obtaining the consent form. WinPepi program was used to calculate the sample size for control and case group based on multiple logistic regression at significant level of 0.05, and 85% power to detect a week association (multiple correlation coefficient, 0.25). The maximum number was for the recessive genotypes of rs16860234 (582 subjects in each group).

**Table 1:** Association of IGF2BP2 polymorphism with GADA negative diabetes among Malaysian subjects and ethnic groups.

IGF2BP2 SNPs	Control		GADA negative diabetes		Recessive	Dominant	Additive
					OR (95% CI)		
	Freq.	11/12/22	Freq.	11/12/22	P-Value		
**rs6777038 (C<T)**							
Combined^*^	0.29	305/258/47	0.33	479/517/104	1.25(0.85–1.85) 0.25	1.27(1.02–1.58) ***0.03***	1.21(1.02–1.44) ***0.03***
Malay^#^	0.28	132/99/20	0.30	202/187/34	0.83(0.44–1.55) 0.55	1.08(0.76–1.54) 0.67	1.01(0.76–1.32) 0.97
Chinese^#^	0.21	123/74/5	0.27	148/129/13	2.39(0.76–7.54) 0.14	1.75(1.16–2.62) ***0.007***	1.69(1.18–2.42) ***0.004***
Indian^#^	0.41	50/85/22	0.41	129/201/57	1.15(0.66–2.00) 0.61	0.97(0.65–1.47) 0.89	1.04(0.78–1.38) 0.80
**rs16860234 (A<C)**							
Combined^*^	0.24	356/210/41	0.30	559/414/124	2.06(1.36–3.12) ***0.001***	1.35(1.09–1.69) ***0.01***	1.36(1.14–1.61) ***0.0004***
Malay^#^	0.22	153/85/12	0.28	224/160/37	2.55(1.19–5.48) ***0.02***	1.40(0.98–2.01) 0.07	1.43(1.07–1.91) ***0.01***
Chinese^#^	0.18	138/53/9	0.24	182/77/30	3.12(1.30–7.49) ***0.01***	1.41(0.92–2.16) 0.11	1.47(1.05–2.05) ***0.02***
Indian^#^	0.36	65/72/20	0.38	153/177/57	1.26(0.71–2.25) 0.43	1.12(0.76–1.65) 0.57	1.12(0.85–1.49) 0.42
**rs7651090 (A<G)**							
Combined^*^	0.32	292/249/67	0.38	452/462/183	1.82(1.30–2.53) ***0.0004***	1.38(1.11–1.71) ***0.004***	1.35(1.16–1.58) ***0.0002***
Malay^#^	0.31	119/107/25	0.35	181/184/55	1.46(0.84–2.56) 0.18	1.39(0.97–1.99) 0.07	1.30(0.99–1.69) 0.06
Chinese^#^	0.22	123/68/9	0.26	166/98/25	2.46(1.07–5.64) ***0.03***	1.34(0.89–2.02) 0.15	1.39(1.01–1.92) ***0.046***
Indian^#^	0.45	50/74/33	0.50	105/180/103	1.56(0.97–2.50) 0.07	1.30(0.86–1.97) 0.22	1.29(0.99–1.69) 0.06

Risk allele is denoted in boldface. *Controlled for age, race, gender and BMI. #Controlled for age, gender and BMI. The outliers (studentized residual is greater than 2.0 or less than −2.0) were excluded Freq., risk allele frequency. 11, homozygous of major allele; 12, heterozygous; 22, homozygous of minor allele. GADA, glutamic acid decarboxylase antibodies.

**Figure 1:**
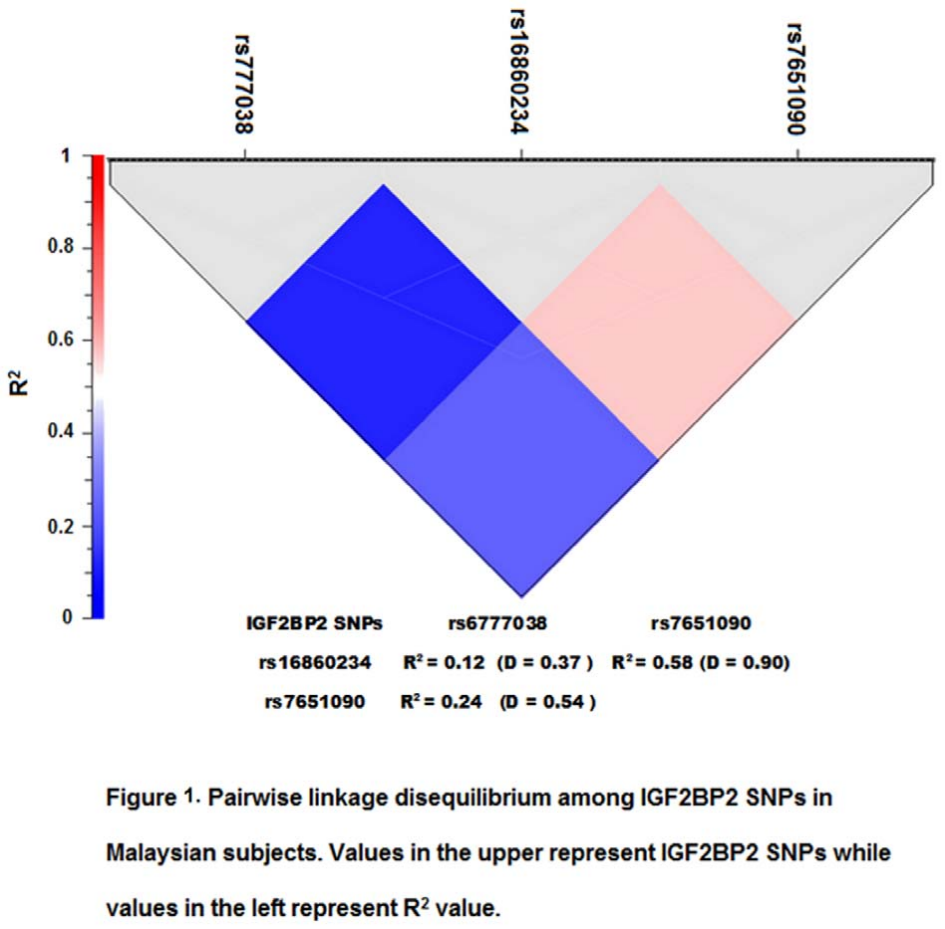
Pairwise linkage disequilibrium among IGF2BP2 SNPs in Malaysian subjects.

### Demographic and Biochemical Analysis

Fasting plasma glucose (FPG), high density lipoprotein cholesterol (HDLc), and triglycerides (TG) were measured by an automated analyzer Dimension® RxL Max® Integrated Chemistry System (Siemens Healthcare Diagnostics Inc., Deerfield, IL USA). Fasting plasma insulin (FPI) was measured by ADVIA Centaur assay XP Immunoassay System (Siemens Healthcare Diagnostics Inc. Deerfield, IL USA). Normal and pathological controls were included with each run of biochemical analysis. GADA were analyzed by ELISA kit RSR GAD65 autoantibody (GAD Ab) (RSR Limited, Cardiff, UK). The analysis was done manually according to the manufacturer’s protocol. Plates were read at 450 nm using a microplate reader (BioRad, Calabasas, USA). GADA Positive and negative controls were included with each run. Blood pressure (BP) and waist parameter were measured using standardized protocols. HOMA-β (a measure of β-cell function) and HOMA-IR (a measure of insulin resistance) were calculated using the Homeostasis Model Assessment (HOMA2) Calculator v2.2, which is available online from Oxford Center for Diabetes, Endocrinology and Metabolism.

**Table 2:** Association of common haplotypes and diplotypes with GADA negative diabetes among Malaysian subjects.

rs6777038,rs16860234, rs7651090	Frequency		P-Value	OR (95% CI)
	Control	GADA negative diabetes		
**Haplotypes**				
TCG	0.26	0.30	0.11	1.22(0.96–1.55)
CAA	0.34	0.26	***0.001***	0.67(0.53–0.84)
CCG	0.13	0.17	***0.01***	1.51(1.10–2.06)
TAA	0.11	0.13	0.29	1.20(0.86–1.67)
TAG	0.10	0.09	0.92	0.98(0.68–1.41)
CAG	0.03	0.03	0.60	1.20(0.61–2.35)
**Diplotypes**				
CAA-CAA	0.34	0.26	***0.001***	0.67(0.53–0.84)
CAA-TCG	0.17	0.17	0.54	0.92(0.69–1.21)
CAA-TAA	0.10	0.12	0.36	1.17(0.84–1.64)
CAA-CCG	0.09	0.10	0.20	1.27(0.88–1.85)
CAA-TAG	0.08	0.07	0.70	0.93(0.62–1.38)
CCG-TCG	0.03	0.04	***0.009***	2.36(1.24–4.47)

Controlled for age, gender, race and BMI. The outliers (studentized residual is greater than 2.0 or less than −2.0) were excluded. GADA, glutamic acid decarboxylase antibodies.

### SNPs Selection and Genotyping

IGF2BP2 alternative variant; rs6777038, rs16860234 and rs7651090 were selected based on [28], [30] studies. The rs7651090 is a tag SNP which is highly linked with the most common IGF2BP2 variants SNPs rs4402960 and rs1470579 [31]. The rs6777038 and rs16860234 are tag SNPs for haplotype blocks among Asians. Genomic DNA was isolated from peripheral blood leukocytes by using a commercially Wizard® Genomic DNA Purification Kit (Promega Corporation, Madison, WI, USA) according to the manufacturer’s protocol. The IGF2BP2 SNPs (rs6777038, rs16860234 and rs7651090) were genotyped by pre-design Taqman genotype assay (Applied Biosystems Inc, Foster City, USA) according to the manufacturer’s protocol using the StepOnePlus Real-Time PCR system (Applied Biosystems Inc, Foster City, USA). The call rates for genotyping were more than 98.5%. To ensure the accuracy, 10% of the samples were re-genotyped; the SNPs genotypes were 100% concordant. No template controls (NTC) were included in each run of real time PCR, which indicated there was no DNA contamination.

**Table 3:** Association of common haplotypes and diplotypes with GADA negative diabetes among Malaysian races.

rs6777038, rs16860234, rs7651090		Control				GADA negative diabetes		
		Malay (n = 254)	Chinese (n = 204)	Indian (n = 162)		Malay (n = 425)	Chinese (n = 293)	Indian (n = 389)
**Haplotypes**								
TCG	Freq.	0.22	0.17		0.42	0.27	0.22	0.39
		OR (95% CI)				1.23(0.81–1.87)	1.62(0.98–2.69)	0.91(0.62–1.34)
		P-Value				0.32	0.06	0.65
CAA	Freq.	0.35	0.44		0.20	0.28	0.37	0.16
		OR (95% CI)				0.65(0.45–0.95)	0.67(0.45–1.01)	0.72(0.44–1.18)
		P-Value				***0.028***	0.06	0.19
CCG	Freq.	0.15	0.10		0.12	0.18	0.11	0.20
		OR (95% CI)				1.49(0.92–2.43)	0.93(0.49–1.78)	2.08(1.16–3.72)
		P-Value				0.11	0.83	***0.01***
TAA	Freq.	0.10	0.13		0.09	0.13	0.16	0.11
		OR (95% CI)				1.21(0.70–2.10)	1.25(0.72–2.18)	1.18(0.62–2.26)
		P-Value				0.49	0.43	0.62
TAG	Freq.	0.11	0.07		0.10	0.09	0.08	0.10
		OR (95% CI)				0.86(0.49–1.51)	1.31(0.60–2.85)	1.07(0.57–2.02)
		P-Value				0.59	0.50	0.84
CAG	Freq.	0.03	0.03		0.02	0.03	0.02	0.03
		OR (95% CI)				2.28(0.74–7.05)	0.58(0.18–1.87)	1.53(0.41–5.65)
		P-Value				0.15	0.36	0.53
**Diplotypes**								
CAA-CAA	Freq.	0.35	0.44		0.20	0.28	0.37	0.16
		OR (95% CI)				0.65(0.45–0.95)	0.67(0.45–1.01)	0.72(0.44–1.18)
		P-Value				***0.028***	0.06	0.19
CAA-TCG	Freq.	0.14	0.13		0.28	0.16	0.13	0.21
		OR (95% CI)				0.99(0.60–1.61)	1.09(0.61–1.94)	0.69(0.45–1.07)
		P-Value				0.96	0.78	0.10
CAA-TAA	Freq.	0.10	0.12		0.08	0.12	0.15	0.10
		OR (95% CI)				1.17(0.67–2.02)	1.16(0.66–2.04)	1.24(0.64–2.42)
		P-Value				0.59	0.60	0.53
CAA-CCG	Freq.	0.11	0.08		0.06	0.14	0.04	0.11
		OR (95% CI)				1.65(0.95–2.87)	0.49(0.21–1.13)	2.45(1.07–5.63)
		P-Value				0.07	0.09	***0.035***
CAA-TAG	Freq.	0.09	0.06		0.07	0.07	0.07	0.08
		OR (95% CI)				0.75(0.40–1.40)	1.33(0.59–2.99)	1.05(0.52–2.12)
		P-Value				0.37	0.50	0.89
CCG-TCG	Freq.	0.02	0.03		0.04	0.04	0.03	0.06
		OR (95% CI)				3.59(1.16–11.09)	1.75(0.52–5.89)	1.96(0.72–5.31)
		P-Value				***0.026***	0.37	0.19

Controlled for age, gender and BMI. The outliers (studentized residual is greater than 2.0 or less than −2.0) were excluded. GADA, glutamic acid decarboxylase antibodies.

### Statistical Analysis

Demographic and biochemical parameters were log-transformed before statistical analyses and then re-transformed back and presented as geometric means. Hardy–Weinberg equilibrium analyses were performed with DeFinetti program (http://ihg.gsf.de/cgi-bin/hw/hwa1.pl) (Institute of Human Genetics). SNP & Variation Suite v7.x (Golden Helix, Bozeman, MT, USA) was used to study the linkage disequilibrium (LD) between SNPs and construct haplotypes and diplotypes of related SNPs. The statistical analyses were conducted using Social Package of Statistical Science (SPSS) 11.5 (LEAD Technologies; Inc. USA). Associations of IGF2BP SNPs, recessive, dominant and additive genetic models, haplotypes and diplotypes with T2D were evaluated by logistic regression controlled for age, gender and body mass index. The significance level was considered P<0.05.

## Results

A total of 1140 patients with T2D and 973 non-diabetic control subjects were included in this study. The GADA analysis on the diabetics revealed 33 GADA positive diabetic patients; (LADA patients) thus; they were excluded from further analysis. Application of the new metabolic syndrome (MetS) criteria [32] on non-diabetic control subjects showed 353 subjects had metabolic syndrome; consequently, they were excluded from subsequent analysis. Hence, 1107 GADA negative diabetes aged 49.5 years (61.6% male and 38.4% female) (target group) and 620 normal subjects without diabetes and MetS aged 51.3 years (67.6% male and 32.4% female) (control group) were recruited in this study. The metabolic and diabetic parameters were significantly different between the target and control groups (Table S1).

The IGF2BP2 SNPs; rs6777038, rs16860234 and rs7651090 did not deviated from Hardy-Weinberg Equilibrium (P-value = 0.49, 0.18, 0.22 in the control group respectively). The logistic regression model (adjusted for age, race, gender and BMI) showed that rs6777038, rs16860234 and rs7651090 were significantly associated with GADA negative diabetes (additive, OR = 1.21; 1.36; 1.35, P = 0.03; 0.0004; 0.0002, respectively) (Table 1). Analysis for the association of these SNPs with diabetes among the three main Malaysian races (Malay, Chinese and Indian) revealed that rs6777038, rs16860234 and rs7651090 were associated with diabetes among Malaysian Chinese (Additive, OR = 1.69; 1.47; 1.39, P = 0.004; 0.02; 0.046, respectively), while only rs16860234 showed a significant association among Malaysian Malay (additive, OR = 1.43, P = 0.01) with diabetes. In Malaysian Indian, the rs7651090 showed a border line association (additive, OR = 1.29, P = 0.06) with diabetes (Table 1).

Three-SNP haplotype and diplotype blocks were identified with significant LD and constructed from the SNPs included in this study (Figure 1). The most common haplotypes and diplotypes are depicted in table 2 (combined races) and in table 3 (three main Malaysian races). The haplotypes and diplotypes with frequency <2% in total sample were excluded from subsequent analysis. The logistic regression model (adjusted for age, race, gender and BMI) showed that the frequency of haplotype CAA was significantly higher in normal subjects compared to diabetic group (OR = 0.67, P = 0.001), particularly in Malay subjects (OR = 0.65, P = 0.028) (Table 2 and 3).

The haplotype TCG containing the risk alleles of the SNPs, showed a borderline risk for diabetes in Chinese (OR = 1.62, P = 0.06). However, the haplotype CCG revealed significant association with diabetes (OR = 1.51, P = 0.01) and this risk of association was higher in diabetic Indian (OR = 2.08, P = 0.01) subjects.

The frequency of diplotype CAA-CAA was higher in normal subjects compared to diabetic subjects (OR = 0.67, P = 0.001) (Table 2), particularly in Malay subjects (OR = 0.65, P = 0.028) (Table 3). The diplotype CCG-TCG was a risk for diabetes (OR = 2.36, P = 0.009) and the association was higher in diabetic Malay (OR = 3.59, P = 0.026) subjects. The diplotype CAA-CCG showed a risk for diabetic Indian (OR = 2.45, P = 0.035) subjects, whereas the other diplotypes had no significant association with T2D.

## Discussion

The association of IGF2BP2 alternative variants; rs7651090, rs16860234 and rs6777038 with GADA negative diabetes was evaluated in Malaysian subjects. To the best of our knowledge, this is the first study to be performed in Southeast Asia. The strongest association was found at rs7651090 followed by rs16860234 then rs6777038. These SNPs showed an association in Malaysian subjects particularly in Chinese. This is in agreement with Hu *et al.* [28] who showed an association of rs7651090 with Chinese and in disagreement with Rong *et al.* [30] who showed no association of rs6777038 and rs16860234 SNPs with Pima Indians. This may be attributed gene–gene and gene–environmental interactions that may have contributed to many of the reported differences in gene–disease associations between different racial or ethnic groups [33]. Another opinion attributed this to ethnic differences and linkage disequilibrium pattern, compounded by the contribution of non-genetic factors and lifestyle changes that can modify the risk of T2D [20].

Several studies suggested the predominant contribution of the IGF2BP2 variants to T2D was mediated through defects in insulin secretion rather than action [34], [35]. However, none of these SNPs showed an association with HOMA-B or HOMA-IR (data not shown). A similar finding was reported in a previous studies [28]. Thus, the effects of these SNPs on diabetes and metabolic syndrome in the Asian might still need further investigations. The haplotype (CCG) and diplotype (CCG-TCG) showed a stronger association with GADA negative diabetes than single individual SNPs. This prompts speculation of possible epistatic interaction between these SNPs in determining overall risk of T2D [20]. This study was a hospital-based and the sampling method was non-probability thus the finding of this study limited its generalization to whole Malaysian population. Sub-grouping of subjects according to races resulted in small sample size particularly in Indian subjects.

In conclusion, IGF2BP2 alternative variants were associated with GADA negative diabetes in Malaysian subjects. The risk of these variants was different among the three main Malaysian races Moreover, certain IGF2BP2 haplotypes and diplotypes strengthen the risk of diabetes.

## Supporting Information

Table S1 **Demography and biochemical parameters.**
(DOCX)
