# Supplementary material for: IGF2BP2 Alternative Variants Associated with Glutamic Acid Decarboxylase Antibodies Negative Diabetes in Malaysian Subjects
Source: PLoS One. 2012 Sep 19;7(9):e45573. doi: 10.1371/journal.pone.0045573 (PMC3446917; doi:10.1371/journal.pone.0045573)
Supplement: Table S1 — Demography and biochemical parameters. (DOCX) [file pone.0045573.s001.docx]

Table S1. Demography and biochemical parameters

| **Parameters** | | **Control (n=620)** | **GADA negative diabetes (n=1107)** | **P-Value** |
| --- | --- | --- | --- | --- |
| Gender% | Male/Female | 61.6/38.4 | 67.6/32.4 |  |
| Races % | Malay | 41.0 | 38.4 |  |
|  | Chinese | 32.9 | 26.5 |  |
|  | Indian | 26.1 | 35.1 |  |
| Age (years) | | 49.5(48.6-50.3) | 51.3(50.8-51.9) | ***0.001*** |
| Body Mass Index (kg/m^2^) | | 24.6(24.3-25.0) | 27.4(27.1-27.7) | ***<0.001*** |
| Waist Circumference (cm) | | 85.2(84.2-86.2) | 96.1(95.4-96.8) | ***<0.001*** |
| Systolic Blood Pressure (mmHg) | | 128(127-130) | 134(133-135) | ***<0.001*** |
| Diastolic Blood Pressure (mmHg) | | 80(79-80) | 81(80-82) | ***0.005*** |
| Fasting Plasma Glucose (mmol/l) | | 4.98(4.94-5.02) | 7.89(7.73-8.05) | ***<0.001*** |
| Fasting Plasma Insulin (pmol/l) | | 59.65(56.35-63.15) | 98.15(94.08-102.39) | ***<0.001*** |
| HOMA-β | | 111.35(107.19-115.67) | 70.84(67.41-74.44) | ***<0.001*** |
| HOMA-IR | | 1.27(1.20-1.35) | 2.45(2.34-2.55) | ***<0.001*** |
| High Density Lipoprotein (mmol/l) | | 1.34(1.32-1.37) | 1.09(1.07-1.10) | ***<0.001*** |
| Triglyceride (mmol/l) | | 1.13(1.09-1.17) | 1.58(1.53-1.63) | ***<0.001*** |

The results presented represent geometric means (95% confidence interval of mean). Blot values are significant. GADA, glutamic acid decarboxylase antibodies
